# Association of early CD4⁺ T-lymphocyte depletion with 28 day mortality in sepsis: a retrospective single-center cohort study

**DOI:** 10.1016/j.clinsp.2026.101173

**Published:** 2026-10-15

**Authors:** Deng Pan, Pin Peng, Yu Fang, Jun Lu, Wendi Zhang, Shanshan Hu, Minghao Fang

**Affiliations:** a Department of Infectious Disease, Xianning Central Hospital, The First Affiliated Hospital of Hubei University of Science and Technology, Xianning, Hubei, China; b Department of Emergency/General Practice, Wuhan University of Science and Technology Affiliated Geriatric Hospital, Wuhan, Hubei, China; c Intensive Care Unit, Tongji Hospital, Tongji Medical College, Huazhong University of Science and Technology, Wuhan, Hubei, China; d Pediatric Intensive Care Unit, Tongji Hospital, Tongji Medical College, Huazhong University of Science and Technology, Wuhan, Hubei, China; e The Second Affiliated Clinical College of Wuhan University of Science and Technology, Wuhan, Hubei, China

**Keywords:** Sepsis, Mortality, CD4⁺ T-lymphocytes, Immune dysregulation, Prognosis, Cohort study

## Abstract

**Introduction:**

Sepsis-induced immune dysregulation, characterized by T-cell loss, is associated with poor outcomes. This study investigated the association between early peripheral T-lymphocyte subset profiles and 28 day mortality in patients with sepsis.

**Methods:**

In this single-center retrospective cohort study, 60 critically ill patients with sepsis and 30 healthy controls were included. Patients with sepsis were classified as 28 day survivors (n = 30) or non-survivors (n = 30). Peripheral T-lymphocyte subset percentages (CD3⁺, CD4⁺, and CD8⁺) were measured by flow cytometry within 24 h of ICU admission.

**Results:**

Compared with healthy controls, patients with sepsis had lower CD3⁺ (55.23±9.15%vs. 68.12±8.04%) and CD4⁺ T-cell percentages (30.88±10.52%vs. 40.15±7.21%) and a lower CD4⁺/CD8⁺ ratio (all p < 0.001). Non-survivors had lower CD3⁺ and CD4⁺ T-cell percentages than survivors (both p < 0.001). In multivariable logistic regression, lower CD4⁺ T-cell percentage (OR = 0.85, 95% CI 0.77–0.94; p = 0.002) and higher APACHE II score (OR = 1.42, 95% CI 1.18–1.71; p < 0.001) were independently associated with 28-day mortality. Adding CD4⁺ T-cell percentage to APACHE II increased the area under the receiver operating characteristic curve from 0.86 to 0.91 (p = 0.03).

**Conclusion:**

Early reduction in peripheral CD4⁺ T-cell percentage was independently associated with 28 day mortality in this selected cohort. CD4⁺ T-cell assessment may provide incremental prognostic information beyond APACHE II, but external validation in larger prospective cohorts is required.

## Highlights

• Early CD4+ T-cell depletion predicts 28-day mortality.
• CD4% inversely correlates with APACHE II and SOFA.
• CD4% and APACHE II are independent predictors.
• Pathogen Gram stain (G+/G−) did not alter CD4% significantly.
• Early flow-cytometry may aid clinical risk stratification.

## Introduction

Sepsis is a life-threatening organ dysfunction caused by a dysregulated host response to infection.^1^ The third international consensus definition (Sepsis-3) defines sepsis as this maladaptive host reaction, which distinguishes it from uncomplicated infection. Globally, sepsis affects millions annually, with high mortality rates, imposing a significant burden on healthcare systems and economies.^2^ Sepsis often progresses to organ impairment, and its incidence and associated mortality continue to rise.^3^ A critical feature of sepsis pathophysiology is a profound state of immune dysregulation that often follows the initial hyperinflammatory phase, predisposing patients to secondary infections and contributing to late mortality.^4^^,^^5^ Therefore, identifying reliable prognostic indicators that reflect this immune dysfunction is crucial for improving clinical management and reducing mortality.

Sepsis-induced immune dysregulation is characterized by a numerical depletion in lymphocyte populations, including CD4⁺ T-cells, CD8⁺ T-cells, B-cells, and NK cells.^6^^,^^7^ Lymphocytes are central to adaptive immunity, mediating cellular and humoral responses.^8^ Advances in flow cytometry have enabled detailed analysis of lymphocyte subsets, revealing their roles in health and disease.^9^ Monitoring lymphocyte subsets shows potential for prognosticating outcomes in sepsis patients.^10^^,^^11^ Previous studies have linked NK cell counts and lymphocyte imbalances to sepsis mortality.^11^^,^^12^ Furthermore, alterations in T-helper lymphocyte subpopulations have been observed across the spectrum of sepsis severity.^13^ Our study aimed to investigate the association between early lymphocyte subset changes and sepsis prognosis. We hypothesized that the extent of early T-cell depletion could predict outcomes and provide information for risk stratification.

## Materials and methods

### Study design and setting

This single-center, retrospective observational study was conducted at the Intensive Care Unit (ICU) of Tongji Hospital, a tertiary care teaching hospital in Wuhan, China. The study was designed and reported in accordance with the Strengthening the Reporting of Observational Studies in Epidemiology (STROBE) guidelines.^14^

### Participants

We retrospectively enrolled 60 critically ill adult patients diagnosed with sepsis between June 2021 and July 2023. A control group of 30 age- and sex-matched healthy volunteers was recruited from hospital staff or their relatives during the same period. Sepsis patients were stratified into two outcome groups based on 28 day mortality: a survival group (n = 30) and a non-survival group (n = 30).

### Eligibility criteria

Inclusion criteria for patients with sepsis were: 1) Diagnosis of sepsis according to the Sepsis-3 criteria and 2) Age 18–95 years. Exclusion criteria were: 1) Major surgery within the preceding 4-weeks 2) Significant trauma; 3) Pre-existing diabetes mellitus; 4) Active malignancy; 5) A known hypersensitivity syndrome; 6) Primary or acquired immunodeficiency, including human immunodeficiency virus infection, long-term immunosuppressive therapy, or corticosteroid use before admission; and 7) Pregnancy.

### Definitions

Sepsis was diagnosed based on the Sepsis-3 guidelines, defined as suspected or confirmed infection plus an acute increase in Sequential Organ Failure Assessment (SOFA) score of ≥ 2 points. Septic shock was defined as sepsis with persisting hypotension requiring vasopressors to maintain a MAP ≥65 mmHg and having a serum lactate level > 2 mmoL/L despite adequate volume resuscitation. Clinical and laboratory indicators supportive of organ dysfunction were also evaluated.

### Determination of infection source

The primary site of infection was determined by a review of clinical, imaging, and microbiological data by two independent intensivists, with discrepancies resolved by consensus.

### Data collection and variables

Data were extracted from electronic medical records. Variables collected included demographic information (age, sex, weight), baseline laboratory values (albumin, C-reactive protein, liver function tests), severity scores (APACHE II, SOFA) calculated on the first day of ICU admission, and microbiological data. The primary exposure variables were the percentages of peripheral T-lymphocyte subsets (CD3⁺, CD4⁺, CD8⁺), and the primary outcome was 28 day all-cause mortality.

### Microbiological analysis

Blood cultures were performed using the BACTEC FX automated system (Becton Dickinson, USA). At least two sets of blood cultures (one aerobic and one anaerobic bottle per set) were drawn from different peripheral sites before the initiation of antimicrobial therapy. True bacteremia was distinguished from contamination based on clinical significance and the recovery of the same organism from multiple blood culture sets. For common skin commensals, such as *Staphylococcus epidermidis*, at least two positive cultures with the same antibiogram were required to be considered pathogenic.

### Assessment of T-cell subsets

Peripheral venous blood samples anticoagulated with EDTA were collected within 24 h of ICU admission for patients with sepsis, before administration of immunomodulatory agents such as corticosteroids, and at a single time point for healthy controls. The percentages of CD3⁺, CD4⁺, and CD8⁺ T-cells within the total lymphocyte population were assessed by flow cytometry using commercially available monoclonal antibodies; antibody specifications are provided in Supplementary Table 1. Flow cytometry was performed using a BD FACSCanto II flow cytometer (BD Biosciences, San Jose, CA, USA), with BD FACSDiva software for data acquisition and FlowJo, version 10, for analysis. Lymphocytes were identified according to forward- and side-scatter properties, followed by singlet gating. CD3⁺ T-cells were gated within the lymphocyte population, and CD4⁺ and CD8⁺ subsets were subsequently identified within the CD3⁺ gate. Fluorescence-minus-one controls were used to define positive-staining gates.

### Statistical analysis

Statistical analyses were performed using SPSS, version 25.0 (IBM Corp., Armonk, NY, USA). An a priori sample-size calculation based on a prior study indicated that 28 patients per outcome group would provide 80% power, at a two-sided α level of 0.05, to detect a 5-percentage-point difference in CD4⁺ T-cell percentage. Accordingly, at least 28 patients were required in each outcome group. Normality was assessed using the Shapiro-Wilk test. Normally distributed continuous variables are presented as mean ± standard deviation and were compared using the independent-samples *t*-test for two groups or one-way analysis of variance with Tukey post hoc testing for three groups. Non-normally distributed continuous variables are presented as median (interquartile range) and were compared using the Mann-Whitney *U test* or Kruskal-Wallis test, as appropriate. Categorical variables are presented as counts (percentages) and were compared using the chi-square test or Fisher exact test. Pearson or Spearman correlation analysis was used, as appropriate. Univariable logistic regression was performed to identify candidate predictors of 28 day mortality. Variables with p < 0.10 in univariable analysis were entered into a multivariable logistic regression model using backward stepwise selection based on the likelihood ratio. Model calibration was assessed using the Hosmer-Lemeshow test, and collinearity was evaluated using variance inflation factors. Receiver operating characteristic curves were used to evaluate discrimination. The areas under the curve for the APACHE II-only model and the combined model containing APACHE II and CD4⁺ T-cell percentage were compared using the DeLong test. Net reclassification improvement and integrated discrimination improvement were calculated to quantify incremental prognostic performance. All tests were two-sided, and p < 0.05 was considered statistically significant. Exact p-values are reported unless p < 0.001.

## Results

### Patient characteristics

A total of 60 patients with sepsis and 30 healthy controls were included. The sepsis cohort was divided into a survival group (n = 30) and a non-survival group (n = 30). Baseline characteristics are shown in Table 1. There were no significant differences in age, weight, or sex distribution between the groups (p > 0.05). However, sepsis patients had significantly lower blood albumin levels (28.81±4.85 g/L vs. 31.72±4.27 g/L, p = 0.004) compared to the control group.

**Table 1:** Baseline characteristics of study participants.

**Parameter**	**Control group (n** **=** **30)**	**Sepsis group (n** **=** **60)**	**Test statistic**	**p-value**
Age (years)	52.38 ± 6.53	53.32 ± 6.21	*t* = −0.715	0.477
Weight (kg)	68.27 ± 12.15	67.95 ± 11.48	*t* = 0.125	0.901
Sex distribution (male/female)	19/11	35/25	χ^2^ = 0.117	0.732
Albumin (g/L)	31.72 ± 4.27	28.81 ± 4.85	*t* = 2.964	0.004

Data are presented as mean ± SD or n. Comparisons were made using independent-samples *t*-test for continuous variables and Chi-Square test for categorical variables.

### Primary infection focus

Among the 60 sepsis patients, respiratory system involvement was the most common site of infection (n = 26, 43.3%), followed by the digestive system (n = 16, 26.7%), urinary system (n = 10, 16.7%), skin and soft tissue (n = 4, 6.7%), and nervous system (n = 4, 6.7%) (Fig. 1).

**Fig. 1:**
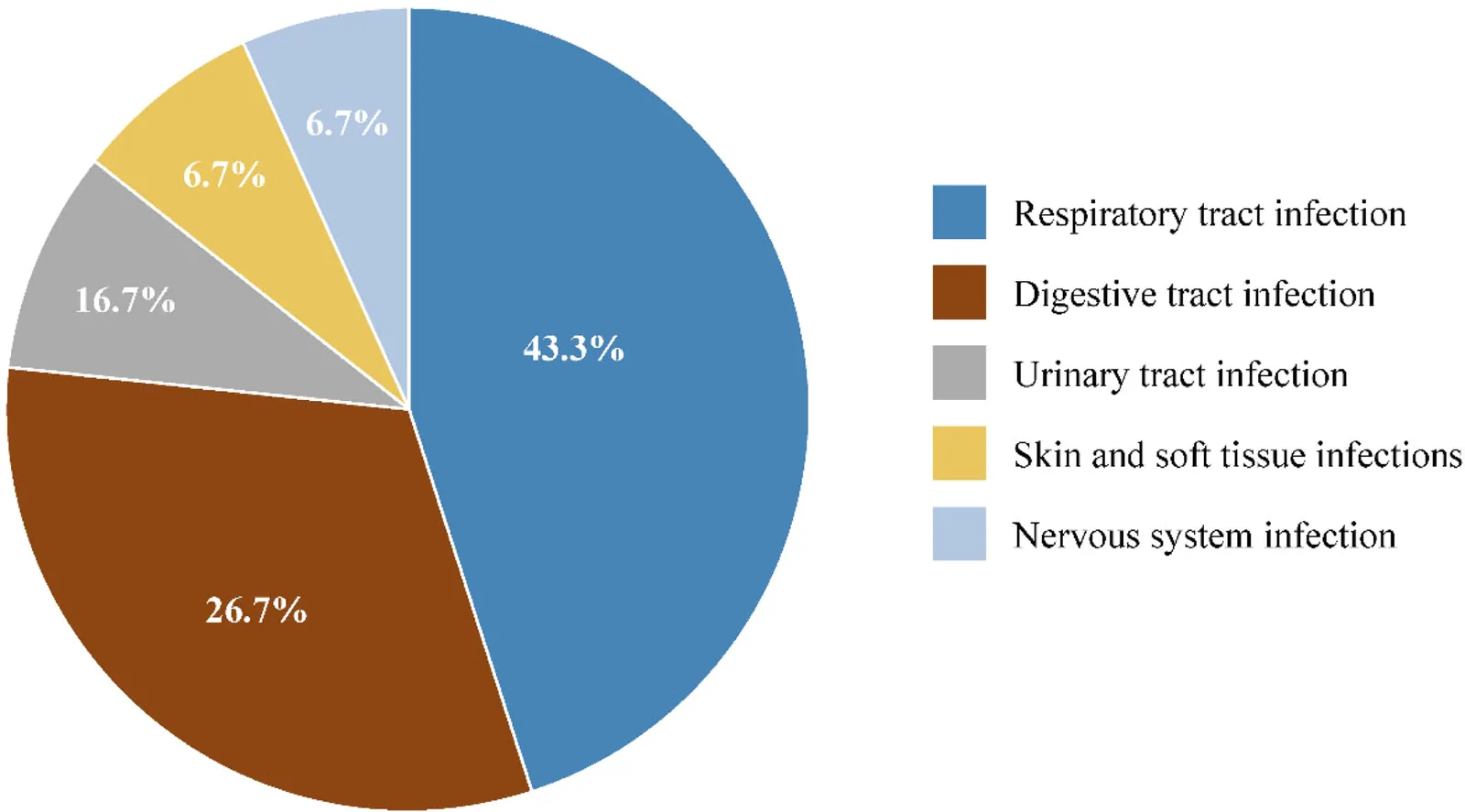
Distribution of primary infection sites in the sepsis cohort (n = 60). The pie chart shows the proportions of cases attributed to the five principal infection sites.

### Blood culture findings

Among the 60 patients with sepsis, blood cultures were positive in 30 cases. Among these, Gram-positive bacteria were predominant (n = 17, 56.7%), while Gram-negative bacteria were identified in 13 cases (43.3%). The distribution is detailed in Table 2. *Staphylococcus epidermidis* was the most prevalent Gram-positive strain, and *Klebsiella pneumoniae* was the most common Gram-negative pathogen.

**Table 2:** Distribution of positive pathogens from blood cultures in sepsis patients (n = 30 positive cultures).

**Pathogen (Gram-positive)**	**Quantity (*n*)**	**Percentage (%)**	**Pathogen (Gram-negative)**	**Quantity (*n*)**	**Percentage (%)**
*Staphylococcus epidermidis*	5	16.7	*Acinetobacter baumannii*	2	6.7
*Staphylococcus haemolyticus*	2	6.7	*Enterobacter cloacae*	1	3.3
*Streptococcus pyogenes*	2	6.7	*Salmonella* spp.	2	6.7
*Staphylococcus hominis*	2	6.7	*Pseudomonas aeruginosa*	2	6.7
*Enterococcus* spp.	1	3.3	*Burkholderia cepacia*	2	6.7
*Staphylococcus aureus*	2	6.7	*Klebsiella pneumoniae*	3	10.0
*Streptococcus agalactiae*	3	10.0	*Escherichia coli*	1	3.3
**Total G+**	**17**	**56.7**	**Total G-**	**13**	**43.3**

### Alterations in T-cell subsets among sepsis patients and controls

As shown in Table 3, groupwise analysis revealed profound alterations in T-lymphocyte subsets. Compared to healthy controls, both the sepsis survival and non-survival groups had significantly lower percentages of CD3⁺ T-cells and CD4⁺ T-cells, as well as a lower CD4⁺/CD8⁺ ratio (all p < 0.001 by ANOVA). Post hoc analysis showed that the non-survival group had a more severe reduction in CD3⁺ T-cells (49.87±7.91%) and CD4⁺ T-cells (24.71±7.82%) compared to both the survival group (60.60±7.82% and 37.05±9.01%, respectively; both p < 0.001) and the control group (p < 0.001). The CD8⁺ T-cell percentage was significantly higher in the non-survival group compared to controls (p = 0.002).

**Table 3:** Comparison of peripheral T-lymphocyte subsets among healthy controls, sepsis survivors, and non-survivors.

**Parameter (%)**	**Control group (n** **=** **30)**	**Sepsis survival group (n** **=** **30)**	**Sepsis non-survival group (n** **=** **30)**	***F*-value**	**p-value**
CD3⁺ T-cells	68.12 ± 8.04	60.60 ± 7.82^a^	49.87 ± 7.91^a^^,^^b^	29.51	<0.001
CD4⁺ T-cells	40.15 ± 7.21	37.05 ± 9.01	24.71 ± 7.82^a^^,^^b^	21.89	<0.001
CD8⁺ T-cells	26.55 ± 6.99	22.18 ± 6.15^a^	29.98 ± 7.50^c^	6.83	0.002
CD4⁺/CD8⁺ ratio	1.51 ± 0.45	1.67 ± 0.61	0.83 ± 0.35^a^^,^^b^	18.76	<0.001

Data are presented as mean ± SD of the percentage of total lymphocytes. One-way ANOVA with Tukey post hoc test was used for comparisons.

a p < 0.05 compared with the Control group.

b p < 0.05 compared with the Sepsis survival group.

c p < 0.05 compared with the Sepsis survival group.

### Comparison of clinical and immunological parameters between sepsis survivors and non-survivors

Table 4 compares the clinical and immunological indices between the sepsis survival (n = 30) and non-survival (n = 30) groups. Non-survivors had significantly higher disease severity scores (APACHE II: 28.78±4.06 vs. 21.43±3.16; SOFA: 12.5 ± 2.8 vs. 7.2 ± 2.1; both p < 0.001), lower albumin levels (25.89±5.11 g/L vs. 31.72±4.27 g/L; p < 0.001), and higher C-reactive protein levels (96.6 ± 10.4 mg/L vs. 38.6 ± 10.1 mg/L; p < 0.001). Immunologically, the non-survival group showed the aforementioned significant reductions in CD3⁺ and CD4⁺ T-cell percentages and the CD4⁺/CD8⁺ ratio.

**Table 4:** Comparison of clinical and immunological indices between sepsis survivors and non-survivors.

**Parameter**	**Sepsis survival group (n** **=** **30)**	**Sepsis non-survival group (n** **=** **30)**	**Test statistic**	**p-value**
Age (years)	52.38 ± 6.53	54.26 ± 5.91	*t* = −1.127	0.265
Sex ratio (male/female)	18/12	17/13	χ^2^ = 0.149	0.700
APACHE II score	21.43 ± 3.16	28.78 ± 4.06	*t* = −7.840	<0.001
SOFA score	7.2 ± 2.1	12.5 ± 2.8	*t* = −8.112	<0.001
Albumin (g/L)	31.72 ± 4.27	25.89 ± 5.11	*t* = 4.821	<0.001
C-reactive protein (mg/L)	38.6 ± 10.1	96.6 ± 10.4	*t* = −21.785	<0.001
AST (U/L)	44.28 ± 11.46	43.16 ± 12.43	*t* = 0.369	0.713
ALT (U/L)	43.95 ± 14.28	40.63 ± 17.38	*t* = 0.781	0.438
CD4⁺ T lymphocyte (%)	37.05 ± 9.01	24.71 ± 7.82	*t* = 5.678	<0.001

Data are presented as mean ± SD or n. Comparisons were made using independent-samples *t*-test for continuous variables and Chi-Square test for categorical variables. APACHE, Acute Physiology and Chronic Health Evaluation; SOFA, Sequential Organ Failure Assessment; AST, Aspartate Aminotransferase; ALT, Alanine Aminotransferase.

### Correlation between CD4⁺ T-cells and disease severity

To further explore the link between T-cell depletion and disease severity, we performed a correlation analysis in the total sepsis cohort (n = 60). The percentage of CD4⁺ T-lymphocytes showed a strong, statistically significant negative correlation with both the APACHE II score (*r* = −0.58, p < 0.001) and the SOFA score (*r* = −0.61, p < 0.001), as illustrated in Fig. 2. This indicates that a greater numerical depletion of CD4⁺ T-cells is associated with higher disease severity.

**Fig. 2:**
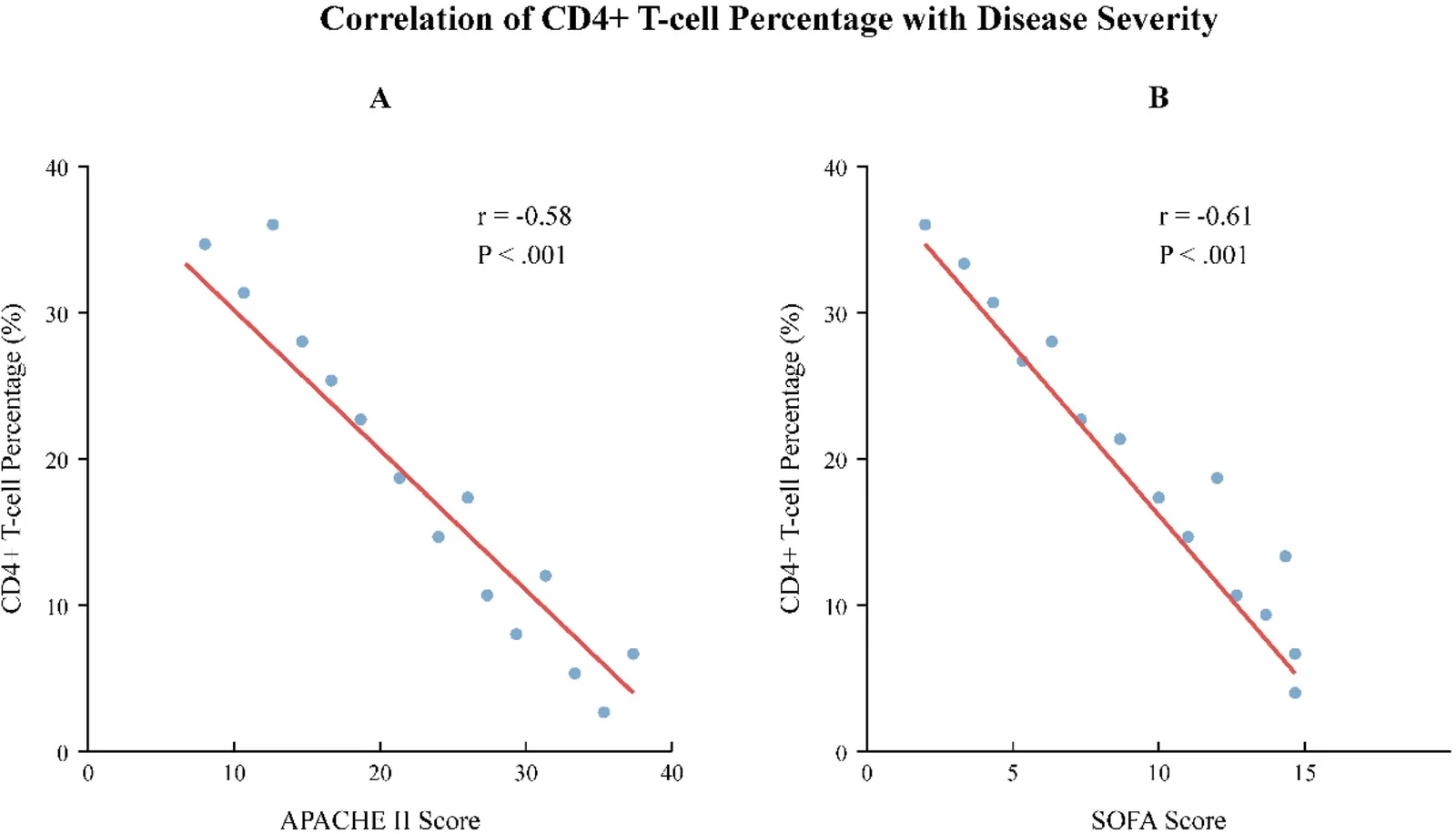
Correlation between CD4⁺ T-cell percentage and disease severity scores in patients with sepsis (n = 60). (A) Correlation between CD4⁺ T-cell percentage and APACHE II score. (B) Correlation between CD4⁺ T-cell percentage and SOFA score. Pearson correlation coefficients (*r*) and p-values are shown.

### Incremental prognostic value of CD4⁺ T-cell percentage

To determine whether measuring CD4⁺ T-cell percentage provided additional prognostic information beyond the standard APACHE II severity score, we compared the predictive performance of two models. The model containing only the APACHE II score demonstrated good predictive ability, with an AUC of 0.86 (95% CI: 0.77–0.95). The addition of the CD4⁺ T-lymphocyte percentage to the model significantly improved its predictive performance, yielding an AUC of 0.91 (95% CI: 0.83–0.98). The comparison between the two models was statistically significant (DeLong test, p = 0.03). Furthermore, the combined model showed improved reclassification, with an NRI of 0.18 (95% CI: 0.02–0.34; p = 0.04) and an IDI of 0.07 (95% CI: 0.01–0.13; p = 0.02), indicating that the inclusion of CD4⁺ T-cell percentage provides a modest but statistically significant enhancement in prognostic accuracy over the APACHE II score alone (Table 5, Fig. 3).

**Table 5:** Incremental prognostic value of CD4⁺ T-lymphocyte percentage for predicting 28-day mortality.

**Model**	**AUC (95% CI)**	**p-value for comparison**^a^	**NRI (95% CI)**	**p-value**	**IDI (95% CI)**	**p-value**
Model 1: APACHE II score	0.86 (0.77–0.95)	0.03	0.18 (0.02–0.34)	0.04	0.07 (0.01–0.13)	0.02
Model 2: APACHE II score + CD4⁺ T-cell percentage	0.91 (0.83–0.98)					

AUC, Area Under the Curve; CI, Confidence Interval; NRI, Net Reclassification Improvement; IDI, Integrated Discrimination Improvement.

a p-value from DeLong test for comparing the AUC of Model 1 and Model 2.

**Fig. 3:**
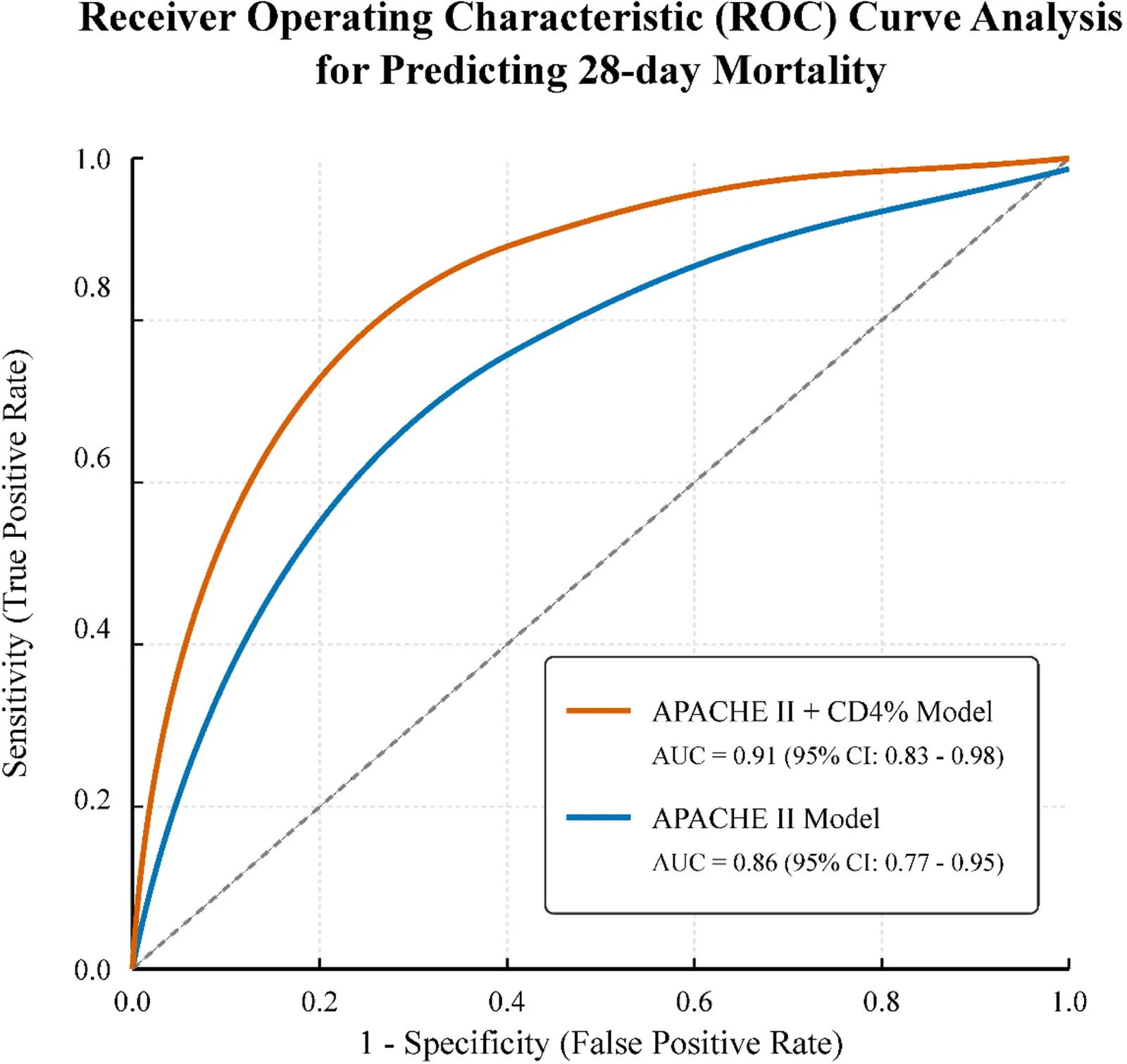
Receiver operating characteristic curve analysis for 28 day mortality. Curves show the discrimination of the APACHE II-only model (AUC = 0.86) and the combined multivariable logistic regression model containing APACHE II score and CD4⁺ T-cell percentage (AUC = 0.91).

### Multivariable analysis of prognostic factors

Variables with p < 0.10 in the between-group analyses shown in Table 4 were entered into a multivariable logistic regression model. The final model identified APACHE II score and CD4⁺ T-lymphocyte percentage as significant independent predictors of 28 day mortality (Table 6). A higher APACHE II score was associated with increased odds of mortality (OR = 1.42; 95% CI: 1.18–1.71; p < 0.001), whereas a higher CD4⁺ T-lymphocyte percentage was associated with decreased odds of mortality (OR = 0.85; 95% CI: 0.77–0.94; p = 0.002). The Hosmer-Lemeshow test indicated adequate model calibration (p = 0.245), and no substantial collinearity was detected (all VIF < 2). The distribution of these two indicators in the non-survival group is shown in Fig. 4.

**Table 6:** Multivariable logistic regression analysis of predictors for 28-day mortality in sepsis patients.

**Variable**	**β**	**Wald**	**p-value**	**Odds ratio (OR)**	**95% confidence interval (95% CI) for OR**
APACHE II score	0.351	12.543	<0.001	1.42	1.18–1.71
CD4⁺ T lymphocyte (%)	−0.161	9.872	0.002	0.85	0.77–0.94

The final model was selected using a backward stepwise (likelihood ratio) method. Hosmer-Lemeshow test p = 0.245. All variables in the final model had a Variance Inflation Factor (VIF) < 2.

**Fig. 4:**
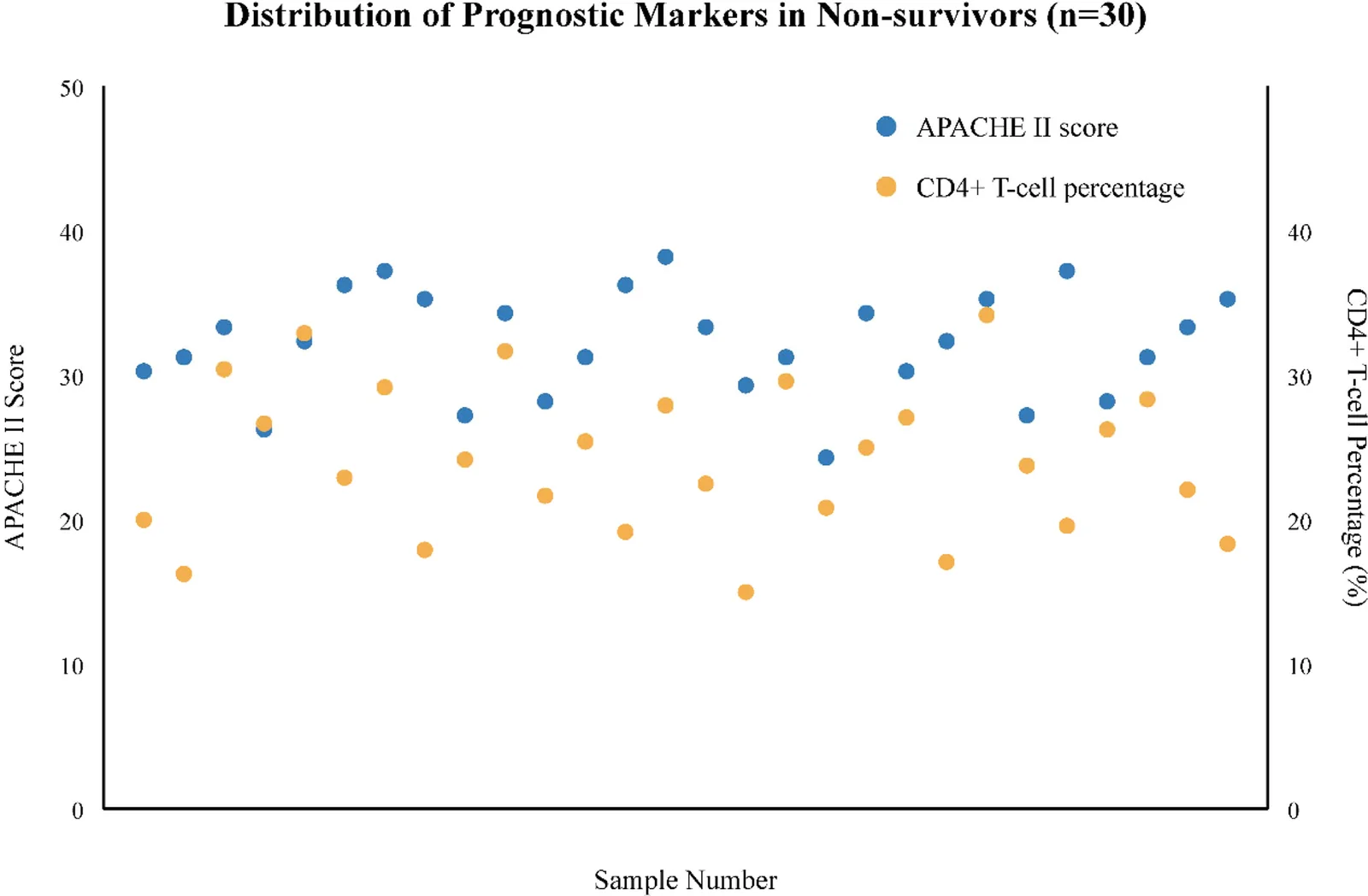
Individual APACHE II scores and CD4⁺ T-cell percentages in the non-survival group (n = 30). Blue circles indicate APACHE II scores, and orange circles indicate CD4⁺ T-cell percentages. The x-axis identifies individual patients.

## Discussion

The pathogenesis of sepsis is complex and involves a dysregulated immune response. Despite advances in management, sepsis-induced immune dysregulation often leads to secondary infections and increased long-term mortality.^15^ In this study, we analyzed 60 sepsis patients and 30 controls and found that APACHE II score and peripheral blood CD4⁺ T-lymphocyte percentage were independent predictors of 28 day mortality. Our results showed that patients who did not survive had significantly higher APACHE II and SOFA scores, and lower albumin and CD4⁺ T-cell percentages, which is consistent with severe disease and profound T-cell depletion.

Lymphocytes are central to adaptive immunity, and their numerical depletion is a hallmark of sepsis-induced immune dysregulation.^7^^,^^16^ CD4⁺ T-cells are critical coordinators of the immune response, and their loss can impair the body's ability to fight infection.^17^ The finding that a lower CD4⁺ T-cell percentage was independently associated with mortality is consistent with studies supporting the prognostic relevance of T-cell lymphopenia in sepsis.^18^^,^^19^ CD4⁺ T-cell percentage was also inversely correlated with APACHE II and SOFA scores, indicating that lower values track with greater illness severity; however, the observational design does not permit causal inference. Adding admission CD4⁺ T-cell percentage to APACHE II produced a statistically significant but modest improvement in prognostic discrimination and reclassification, suggesting that this immune marker may provide information not fully captured by physiological severity scoring.

Pathogen-specific differences in immune status were not evaluated because the numbers of culture-positive cases within individual microbial categories were insufficient for reliable comparative analysis. The relationship between pathogen class and host immune responses is complex, and determining whether distinct microbial groups are associated with different patterns of T-cell depletion will require larger datasets.^20^

This study has several limitations. First, its single-center, retrospective design and strict exclusion criteria ‒ particularly the omission of patients with common comorbidities like diabetes mellitus and active malignancy ‒ limit the generalizability of our findings. These conditions are prevalent in the ICU and are associated with baseline immune alterations, meaning our cohort is not fully representative of the broader sepsis population. Our results should therefore be interpreted as applicable to a selected subgroup of sepsis patients without these major pre-existing immune-modulating conditions. Second, the sample size of 60 patients, with 30 mortality events, is modest for multivariable logistic regression. Although the events-per-variable ratio for our final model is 15, which meets the minimum threshold of 10, the model's stability could be limited, and the risk of overfitting cannot be excluded. Larger, multicenter prospective studies are needed to validate our findings and develop a more robust predictive model. Third, we did not control for all potential confounding variables, such as the specifics and timing of antimicrobial therapy, source control procedures, or other supportive care measures, which could influence both immune status and outcomes. Fourth, we measured only the percentages of T-cell subsets, which indicates numerical depletion rather than functional immunosuppression. A comprehensive immune assessment would also include functional assays (e.g., cytokine production capacity, expression of exhaustion markers like PD-1) to fully characterize the state of immune paralysis.^21^^,^^22^ Fifth, lymphocyte subsets were measured only once upon ICU admission. This provides a static snapshot of a dynamic process. The trajectory of T-cell percentages (i.e., persistent depletion versus recovery) may hold greater prognostic power than a single baseline value, a concept well-supported in recent literature.^23^, ^24^, ^25^ Future prospective studies should incorporate serial monitoring of T-cell subsets to better understand the dynamic immune response and its relationship with clinical outcomes in sepsis.

## Conclusion

In this selected cohort of patients with sepsis, a lower peripheral blood CD4⁺ T-cell percentage at ICU admission was independently associated with 28 day mortality alongside the APACHE II score. Adding CD4⁺ T-cell percentage to APACHE II provided a modest improvement in prognostic performance. Early CD4⁺ T-cell assessment may therefore serve as an adjunct to conventional risk stratification, although validation in larger, prospective, multicenter cohorts is required.

## Ethics approval and consent to participate

The study was conducted in accordance with the Declaration of Helsinki and was approved by the Ethics Committee of Tongji Hospital, Tongji Medical College, Huazhong University of Science and Technology (approval n° TJ-IRB202309052). Written informed consent was obtained from all participants or their legally authorized representatives. For critically ill patients unable to provide consent, consent was obtained from a legally authorized representative.

## Consent for publication

Not applicable.

## Authors’ contributions

DP and PP conceived and designed the study. YF and JL performed the experiments. WZ, SH, and MF collected and analyzed the data. DP wrote the initial draft of the manuscript. PP provided critical revision of the manuscript for important intellectual content. All authors reviewed and approved the final version of the manuscript.

## Funding

This research did not receive any specific grant from funding agencies in the public, commercial, or not-for-profit sectors.

## Declaration of competing interest

The authors declare no conflicts of interest.

## Data Availability

The anonymized dataset generated and analyzed during the current study is available from the corresponding author on reasonable request.
