# Supplementary material for: Association of early CD4⁺ T-lymphocyte depletion with 28 day mortality in sepsis: a retrospective single-center cohort study
Source: Clinics (Sao Paulo). 2026 Oct 15;81:101173. doi: 10.1016/j.clinsp.2026.101173 (PMC13693353; doi:10.1016/j.clinsp.2026.101173)
Supplement: Supplementary file 1 [file mmc1.docx]

CLINICS-D-25-01793_Supplementary Material

**Supplementary Table 1** Details of antibodies used for flow cytometry

| **Target** | **Fluorochrome** | **Clone ID** | **Manufacturer** | **Catalog Number** |
| --- | --- | --- | --- | --- |
| CD3 | PE-Cy5 | UCHT1 | BD Biosciences | 555334 |
| CD4 | FITC | RPA-T4 | BD Biosciences | 555346 |
| CD8 | PE | RPA-T8 | BD Biosciences | 555367 |
